# WSB1, as an E3 ligase, restrains myocardial ischemia–reperfusion injury by activating β-catenin signaling via promoting GSK3β ubiquitination

**DOI:** 10.1186/s10020-024-00800-3

**Published:** 2024-02-23

**Authors:** Lini Fang, Yang Tao, Guoying Che, Yongzi Yun, Min Ren, Yujie Liu

**Affiliations:** 1 Department of Function, Sanya Central Hospital (Hainan Third People’s Hospital), 1154# Jiefang Fourth Road, Sanya, Hainan Province China; 2 Department of Ultrasound Medicine, The Second Affiliated Hospital of Harbin Medical University, Harbin, Heilongjiang Province China; 3 Ultrasound Department, Shanghai First Maternity and Infant Hospital, School of Medicine, Tongji University, 536# Changle Road, Shanghai, China

**Keywords:** Myocardial ischemia–reperfusion, WSB1, β-catenin, GSK3β, Ubiquitination

## Abstract

**Background:**

Reperfusion is the most effective strategy for myocardial infarct, but induces additional injury. WD repeat and SOCS box containing protein 1 (WSB1) plays a protective role in ischemic cells. This study aims to investigate the effects of WSB1 on myocardial ischemia–reperfusion (IR) injury.

**Methods:**

The myocardial IR was induced by left anterior descending (LAD) ligation for 45 min and subsequent reperfusion. The overexpression of WSB1 was mediated by tail vein injection of AAV9 loaded with WSB1 encoding sequence two weeks before IR surgery. H9c2 myocardial cells underwent oxygen-sugar deprivation/reperfusion (OGD/R) to mimic IR, and transfected with WSB1 overexpression or silencing plasmid to alter the expression of WSB1.

**Results:**

WSB1 was found highly expressed in penumbra of myocardial IR rats, and the WSB1 overexpression relieved IR-induced cardio dysfunction, myocardial infarct and pathological damage, and cardiomyocyte death in penumbra. The ectopic expression of WSB1 in H9c2 myocardial cells mitigated OGD/R-caused apoptosis, and silencing of WSB1 exacerbated the apoptosis. In addition, WSB1 activated β-catenin signaling, which was deactivated under the ischemic condition. The co-immunoprecipitation results revealed that WSB1 mediated ubiquitination and degradation of glycogen synthase kinase 3 beta (GSK3β) as an E3 ligase in myocardial cells. The effects of WSB1 on myocardial cells under ischemic conditions were abolished by an inhibitor of β-catenin signaling.

**Conclusion:**

WSB1 activated β-catenin pathway by promoting the ubiquitination of GSK3β, and restrained IR-induced myocardial injury. These findings might provide novel insights for clinical treatment of myocardial ischemic patients.

**Supplementary Information:**

The online version contains supplementary material available at 10.1186/s10020-024-00800-3.

## Introduction

Ischemic heart disease (IHD) is the leading cause of death in the world (Finegold et al. 2013). Acute myocardial infarction is the most severe manifestation of IHD, impacting 800,000 people every year, with a mortality of 27% (Boateng and Sanborn 2013). The disruption of a vulnerable atherosclerotic plaque or erosion of the coronary artery endothelium causes myocardial infarction, which is characterized by cardiomyocyte death (Reed et al. 2017). Timely reperfusion by means of primary percutaneous coronary intervention is the most effective strategy to restore oxygen supply and maintain cardiac function. However, the blood reperfusion induces further cardiomyocyte death, a phenomenon known as myocardial reperfusion injury (Braunwald and Kloner 1985; Hausenloy and Yellon 2013). It is important for patients with acute myocardial infarction to minimize reperfusion injury during rescue.

The analysis from databank Gene Expression Omnibus (GEO) (https://www.ncbi.nlm.nih.gov/geo/) shows that in rodents with myocardial ischemia–reperfusion (IR) treatment, WD repeat and SOCS box containing protein 1 (WSB1) is highly expressed in myocardial tissues (GSE160516, GSE4105). WSB1 is an E3 ligase, mediates ubiquitination and degradation of substates, such as ataxia-telangiectasia mutated (ATM), leucine-rich repeat kinase 2 protein (LRRK2) and von Hipple-Lindau tumor suppressor (VHL) (Kim et al. 2017, 2015; Nucifora et al. 2016). Ubiquitination-proteasome degradation pathway is a conserved and dominant protein degradation pathway in eukaryotic cells. Briefly, the obsolete, damaged or misfolded proteins are marked by ubiquitin, recognized by receptors containing ubiquitin-binding domains and degraded by 26S proteasome (Chen et al. 2021; Husnjak and Dikic 2012). The binding of ubiquitin to substrates is mediated by three enzymes: activating enzyme (E1), conjugating enzyme (E2) and ligase (E3). Ubiquitin was activated by E1 and transferred onto E2, and E3 ligase interacts with ubiquitin-E2 complex and substrate to mediate the binding between ubiquitin and substrate (Hershko et al. 2000). E3 ligase determines the specificity of substrates. Acting as an E3 ligase, WSB1 was reported to play a protective role in tumor cells and non-tumor cells by catalyzing the ubiquitination and proteasomal degradation of substrate molecules (Nucifora et al. 2016; Kim et al. 2015).

The analysis from bioinformatics website HitPredict (http://www.hitpredict.org/) displays that WSB1 directly interacts with glycogen synthase kinase 3 beta (GSK3β), and may mediate its ubiquitination. GSK3β is a kinase involving Wnt/β-catenin signaling. In the inactive state, β-catenin is phosphorylated by destruction complex composed of axin, adenomatous polyposis coli (APC), casein kinase 1 (CK1) and GSK3β, followed with ubiquitination and degradation. When this signaling is activated by wnt ligand or other factors, the destruction complex is degraded, and β-catenin continuously expresses and transfers into nuclei to activate the transcription of target genes (Shang et al. 2017; Nusse and Clevers 2017; Pai et al. 2017). Wnt/β-catenin signaling has been reported with a protective effect in myocardial IR injury (Chen et al. 2020; Yan et al. 2020). Another member of WSB subfamily, WSB2, was demonstrated to activate Wnt/β-catenin signaling (Wei et al. 2020). We assumed that WSB1 may possess a similar regulatory effect on this signaling. In addition, WSB1 is transcriptionally activated by hypoxia inducible factor (HIF)-1, and also stabilizes HIF-1 protein molecule in tumor cells with hypoxic condition. This positive feedback loop plays a protective role in hypoxic cells (Kim et al. 2015; Tong et al. 2013). However, the function of WSB1 in ischemic diseases has not been elucidated. Here, we speculated that WSB1 may play a protective role in myocardial ischemia and regulate β-catenin signaling by mediating GSK3β degradation.

In our previous reports, calycosin-7-O-β-D-glucoside (CG) was demonstrated with beneficial effects on maintaining cardiac function and cardiomyocyte survival in rodents after IR surgery (Ren et al. 2016; Liu et al. 2020). Subsequently, we found that the CG treatment led to further upregulation of WSB1, which suggested the potential myocardial protective role of WSB1 in IR-treated animals. In this study, we try to investigate that 1) whether WSB1 alleviated the myocardial IR injury in rats; 2) whether and how WSB1 regulated Wnt/β-catenin signaling.

## Material and methods

### Bioinformatics analysis

We searched “WSB1” and “myocardial ischemia reperfusion” in GEO databank. GSE160516 dataset showed the GeneChip array of mouse heart after IR for 6 h, 24 h or 72 h. GSE4105 dataset exhibited the transcriptome analysis of left ventricles from rats with left anterior descending (LAD) ligation for 30 min followed with reperfusion for 2 days or 7 days. The expression of WSB1 in GSE160516 and GSE4105 was analyzed.

### Animal model

Healthy male SD rats (8–10 weeks old, 250–300 g) were purchased from ChangSheng Biotech. (Benxi, China), and kept in a controlled environment (12 h light/12 dark cycles at 22 ± 1 ℃ with a humidity of 45–55%) with accesses to food and water. The experimental procedure was lined with Guide for the Care and Use of Laboratory Animals (8th, NIH), and approved by Ethics Committee of Sanya Central Hospital (Approval ID: TJLAC-020-154).

The myocardial IR was induced according to previously reported (Fan et al. 2019). Briefly, the LAD coronary artery was ligated at the site of its emergence from the left atrium using a nylon suture with a slipknot. After ischemia for 45 min, the occlusion was removed, followed with reperfusion of 6 h, 24 h and 72 h. The sham animals underwent the same surgical procedures without tying around the LAD coronary artery.

The calycosin-7-O-β-D-glucoside (CG) (30 mg/kg; Macklin, Shanghai, China) was intravenously injected into rats 45 min prior to ligation of the LAD coronary artery according to our previous report (Liu et al. 2020). After reperfusion for 24 h, the rats were subjected to euthanasia.

To change the expression of WSB1, the AAV9 virus carrying WSB1 coding sequence (CDS) (2 × 10^11 vg/200 μl) was delivered into rats via vein injection two weeks before myocardial IR surgery.

### Echocardiograghy

After 24 h reperfusion, the cardiac functions of rats were measured using an animal ultrasonic instrument (VisualSonics, Toronto, Canada). The left ventricular end diastolic diameter (LVEDD), left ventricular systolic diameter (LVESD), left ventricular fraction (LVEF) and fractional shortening (FS) were calculated.

### ELISA

After reperfusion, the blood was collected from carotid artery. The serum levels of creatine kinase MB isoenzyme (CK-MB) and cardiac troponin I (cTnI) were detected using a rat CK-MA ELISA kit or a rat cTnI ELISA kit (FineTest, Wuhan, Hubei, China) according to the manufacturer’s protocols.

### Lactate dehydrogenase (LDH) activity determination

The LDH activity in serum was determined with a LDH assay kit (Jiancheng, Nanjing, Jiangsu, China) according to the manufacturer’s instruction.

### Triphenyltetrazolium chloride (TTC) staining

After euthanasia, the heart was isolated from rat and frozen at -20 ℃ for 10 min. Then the heart tissues were made into 2-mm sections, which were stained with 1% TTC reagent (Solarbio, Beijing, China) at 37 ℃ in the dark for 10 min. After turn over, the sections were repeatedly stained for 10 min, and the myocardial infarction size was assessed according to the pale region.

### Hematoxylin&eosin (H&E) staining

The myocardial tissues were made into conventional paraffin sections with 5 μm, which were subjected to deparaffinization with xylene and grading concentrations of ethanol. Subsequently, the sections were stained with hematoxylin (Solarbio) for 5 min, followed with differentiation with 1% hydrochloric acid/ethanol for 3 s. After rinsing with water, the section were counterstained with eosin (Sangon Biotech (Shanghai) Co., Ltd., Shanghai, China) for 3 min, and dehydrated with ethanol and xylene. Finally, the sections were sealed with neutral gum, and photographed with a microscope at a 200 × magnification.

### Immunohistochemistry staining

The myocardial paraffin sections were made using the routine method. After deparaffinization with xylene for 15 min twice and ethanol of different concentrations (100% for 5 min twice, 95% for 2 min, 85% for 2 min, and 75% for 2 min), the sections were incubated with antigen retrieval buffer in boiling for 10 min. After rinsing with PBS, the sections were blocked with 3% H_2_O_2_ and 1% bovine serum albumin (BSA) (Sangon Biotech (Shanghai) Co., Ltd.) for 15 min, respectively. Incubation with antibody against WSB1 (1:100; Novus Biologicals, Littleton, CO, USA) was performed in a humid box at 4 ℃ overnight, followed with the incubation with HRP-labeled IgG (1:500; Thermo Scientific, Pittsburgh, PA, USA) at 37 ℃ for 60 min. Afterwards, the sections were interacted with DAB color developing agent (MXB biotechnologies, Fuzhou, Fujian, China), followed with counterstaining with hematoxylin and differentiation with 1% hydrochloric acid/ethanol. Finally, the sections were re-dehydrated and sealed. The images were taken at a 400 × magnification.

### Cell culture

Rat myocardial cell H9c2 was purchased from iCell (Shanghai, China), and cultured with DMEM (Procell, China) supplemented with 10% fetal bovine serum (FBS) (Procell) in a humid incubator of 37 ℃ and 5% CO_2_.

For transfection, the cells were incubated with plasmid in the presence of Lipofectamine3000 reagent (Invitrogen) at 37 ℃.

To ectopic expression or silencing of rat WSB1, the WSB1 CDS was cloned into pcDNA3.1-flag vector (YouBio, Changsha, Hunan, China), and short hairpin fragment targeting 5’-GAGTCGTTGTGTTAATATAGA-3ʹ site in WSB1 CDS was inserted into pRNAH1.1 vector. The GSK3β wild type (GSK3β^WT^) or mutant with Y216 to phenylalanine (F) (GSK3β^Y216F^) coding sequence was cloned into pcDNA3.1-HA vector (YouBio) for investigating the function of Y216 in GSK3β molecule.

To simulate the ischemia in vivo, the H9c2 cells were subjected to an oxygen-sugar deprivation (OGD) treatment. Briefly, the cells were cultured with sugar-free and serum-free medium in a hypoxic condition (95% N_2_ and 5% CO_2_) at 37 ℃ for 6 h, and then oxygen and sugar supply were recovered for 4 h or 24 h to mimic the reperfusion (R).

The cells were treated with CG (1 μM) (Macklin) for 24 h to investigate the effects of CG on expression of WSB1.

To interdict the translations of proteins, cycloheximide (CHX) (100 μg/ml) (MedChemExpres, Monmouth Junction, NJ, USA) was used to treat the cells for 0 h, 3 h, 6 h, 9 h 12 h or 15 h.

To intercept the proteasome degradation, MG132 (10 μM) (Aladdin, Shanghai, China) was applied in H9c2 cells for 9 h.

An inhibitor of β-catenin pathway, PNU74654 (30 μM) (Aladdin), was used to disturb the transcriptional activation function of β-catenin in H9c2 cells.

### Western blot

The total protein was extracted from myocardial tissues or H9c2 cells using RIPA lysis buffer with 1% PMSF (Solarbio) on the ice. After centrifugation at 10000 g at 4 ℃ for 5 min, the supernatant was isolated as the protein sample. After concentration determination using a BCA protein assay kit (Solarbio), and denaturation by boiling for 5 min, the protein was separated with SDS-PAGE at 80 V, and the gel concentration varied depending on the protein size. The protein in the gel was then transferred onto PVDF membrane (Millipore, Billerica, MA, USA), which was cut horizontally according to the protein size. Then the membrane was blocked with 5% skim milk (Sangon Biotech, Shanghai, China) or 5% BSA, and incubated with the following antibodies at 4 ℃ overnight: rabbit anti-cleaved caspase-3 (1:1000; Affinity Biosciences, Changzhou, China), rabbit anti-caspase-9 (1:1000; ProteinTech, Rosemont, IL, USA), rabbit anti-Bax (1:1000; ABclonal, Wuhan, China), rabbit anti-Bcl-2 (1:500; ABclonal), rabbit anti-Wnt1 (1:500; Affinity Biosciences), rabbit anti-β-catenin (1:500; ABclonal), rabbit anti-non-phospho (active) β-catenin (1:500; ABclonal), rabbit anti-WSB1 (1:1000; ThermoFisher SCIENTIFIC, Pittsburgh, PA, USA), rabbit anti-GSK3β (1:5000; ProteinTech), rabbit anti-GSK3β^S9^ (1:1000; ABclonal), rabbit anti-GSK3β^Y216^ (1:500; Affinity Biosciences), mouse anti-GAPDH (1:10000; ProteinTech) and rabbit anti-histone H3 (1:1000; GeneTex, Irvine, CA, USA). After washing with TBST, the membrane was incubated with HRP-labeled goat anti-rabbit IgG or anti-mouse IgG (1:3000; Solarbio) at 37 ℃ for 60 min. Subsequently, the membrane interacted with ECL reagent (Solarbio), followed with a signal exposure in the dark. The phosphorylation antibodies were diluted in 5% BSA, and other antibodies diluted in 5% skim milk. The optical density of the banks was analyzed using Gel-Pro-Analyzer software.

### Immunofluorescence staining

The rats were intraperitoneally injected with Evans blue dye (EBD) reagent (10 mg/ml) 14 h prior to IR injury. After 24 h reperfusion, the myocardial tissues were isolated for fixation with 4% paraformaldehyde for 24 h, dehydration with 20–30% sucrose solution for 24–48 h, and embedding with OCT. Then the tissues were frozen and cut into 10-μm sections, which suffered from antigen retrieval in boiling for 10 min and blocking with 1% BSA for 15 min. Subsequently, the sections were incubated with rabbit antibody against Caveolin-3 (CaV3) (Abcam, Cambridge, UK) at a 1:100 dilution at 4 ℃ in the dark overnight, rinsed with PBS, and incubated with FITC-labeled goat anti-rabbit IgG (Abcam) at 1:200 at the temperature for 60 min. Finally, the nuclei were stained with DAPI, and the sections were sealed with anti-fading reagent (Solarbio), and photographed with a fluorescence microscope (Olympus, Tokyo, Japan) at a 400 × magnification.

The H9c2 cells were pre-seeded on the glass slide. After culture for a period of time, the cells on the slide were fixed with 4% paraformaldehyde (Sinopharm, Shanghai, China) at room temperature for 15 min, and permeated with 0.1% TritonX-100 (Beyotime Biotech, Shanghai, China) for 30 min. After blocking with 1% BSA, the cells were incubated with rabbit antibody against β-catenin (1:100; ABclonal) at 4 ℃ overnight, rinsed with PBS, and incubated with Cy3-labeled goat anti-rabbit IgG (1:200; Invitrogen, Carlsbad, California, USA) at room temperature for 60 min. Finally, the cells were mounted with anti-fading reagent (Solarbio). The images were taken with a fluorescence microscope (Olympus, Tokyo, Japan) at a 400 × magnification.

### TUNEL assay

The myocardial tissues were made into paraffin sections, followed with deparaffinization with xylene and ethanol (xylene for 15 min twice, absolute ethanol for 5 min twice, 95%, 85% and 75% ethanol for 2 min, respectively). After permeating with 0.1% TritonX-100 for 8 min at room temperature, the sections were incubated with TUNEL reagent (prepared when using) in a humid and lucifugal box at 37 ℃ for 60 min. Finally, the sections were counterstained with DAPI in the dark for 5 min, and sealed with anti-fading reagent (Solarbio). The images were photographed at a 400 × magnification.

### Real-time PCR

Total RNA was isolated from myocardial tissues or H9c2 cells using a TRIpure total RNA extraction kit (BioTeke Corporation Co., Ltd., Beijing, China) according to the manufacturer’s instruction. After concentration determination using a NANO2000 ultraviolet spectrophotometer (Thermo Scientific), the RNA was reversely transcribed into cDNA using BeyoRT II M-MLV reverse transcriptase (Beyotime Biotech, Shanghai, China) with Oligo(dT) and random primer as the primers. The quantitative PCR was carried out to measure the expression of WSB1, cyclin D1 and c-MYC using the cDNA as template, in the presence of specific primers, fluorochrome SYBR GREEN (Solarbio) and 2 × Taq PCR MasterMix containing dNTP (Solarbio). The PCR procedure was set as follows: pre-denaturation at 95 ℃ for 5 min 10 s, annealing at 60 ℃ for 10 s, extension at 72 ℃ for 15 s, followed with 40 cycles of 72 ℃ for 1 min 30 s, 40 ℃ for 1 min, 60–94 ℃ every 1 ℃ for 1 s, and 25 ℃ for 1 min. The PCR procedure was performed with an Exicycler™ 96 V4 real-time quantitative thermal block (Bioneer, Daejeon, Korea), and the data was calculated using a 2^−ΔΔCT^ method. The primers were synthesized by General Biology Co., Ltd. (Chuzhou, Anhui, China), and the sequence information was shown as follows: WSB1 forward, 5ʹ-TTCAGTTGGAGCCAGTAA-3ʹ, reverse, 5ʹ-CGTATCGTAAGACGCAGTA-3ʹ; cyclin D1 forward, 5ʹ-GCAGAAGTGCGAAGAGG-3ʹ, reverse, 5ʹ-GGCGGATAGAGTTGTCAGT-3ʹ; c-MYC forward, 5ʹ-ATGATGACCGAGCTACTTG-3ʹ, reverse, 5ʹ-GCTGGTGCTGTCTTTGC-3ʹ.

### CCK-8 assay

The H9c2 cells were cultured in 96-well plates, and subjected to certain treatments. CCK-8 reagent (KeyGEN BioTECH, Nanjing, Jiangsu, China) was applied to treat cells with 10 μl per well for 2 h. Then the optical density of supernatant was determined with a microplate reader (BioTek, Winooski, VT, USA) at 450 nm.

### Flow cytometry

The cells were collected for washing with PBS, and centrifuged at 800 g for 5 min. The cells in the precipitate were resuspended with binding buffer, and stained with Annexin V-FITC and propidium Iodide (PI) (KeyGEN BioTECH) at room temperature in the dark for about 10 min. Subsequently, the apoptosis was analyzed using a flow cytometer (ACEA, San Diego, USA).

### Co-immunuprecipitation (Co-IP)

Co-IP was performed to confirm the interaction between two proteins. The cellular protein was extracted from H9c2 cells using RIPA lysis buffer supplemented with 1% PMSF (Beyotime Biotech). After protein concentration determination, Co-IP was performed using a Co-IP kit (Thermo Scientific) according to the manufacturer’s instruction. Briefly, the antibody against GSK3β (ProteinTech) or flag tag (ProteinTech) was immobilized to AminoLink resin by incubating in sodium cyanoborohydride solution (75 mM), and the resin was pre-cross-linked to spin column. The protein samples were incubated with the antibody-resin complex in the tube for immunoprecipitation. Subsequently, the immunoprecipitate was eluted with elution buffer, and used for western blot to detect the content of GSK3β, WSB1, ubiquitin and GAPDH. The information of antibodies was shown as follows: rabbit anti-GSK3β (1:5000; ProteinTech), rabbit anti-WSB1 (1:1000; Thermo Scientific), rabbit anti-ubiquitin (1:5000; ProteinTech), mouse anti-flag tag (1:5000; ProteinTech), rabbit anti-HA tag (1:5000; ProteinTech), goat anti-rabbit IgG-HRP (1:3000; Solarbio), goat anti-mouse IgG-HRP (1:3000; Solarbio).

### Statistical analysis

The data in this study was presented as mean ± SD in six (in vivo) or three individuals (in vitro), and analyzed using Graphpad software. The data in two independent groups was compared with student t test, and data in multiple groups was analyzed using one-way ANOVA followed with Bonferroni’s multiple comparisons. A p value less than 0.05 was considered as statistically significant. *p < 0.05, **p < 0.01, ***p < 0.001.

## Results

### WSB1 was highly expressed in rodents with experimental myocardial IR treatment.

**Fig. 1:**
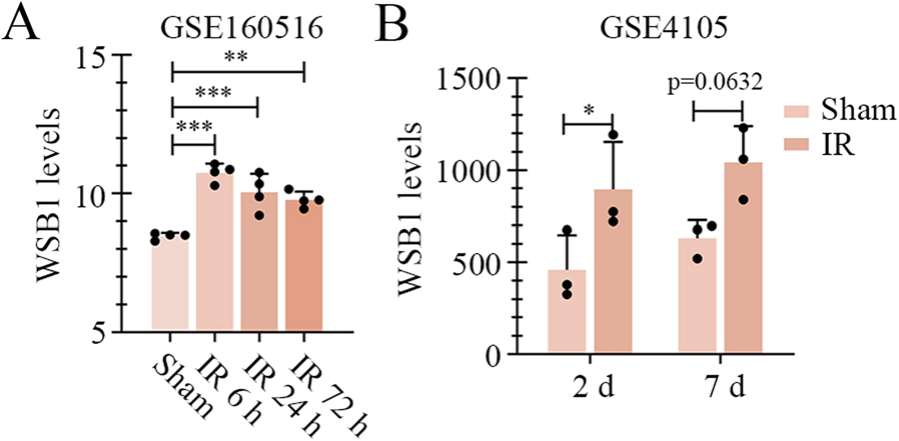
WSB1 was highly expressed in rodents with experimental myocardial IR treatment in GEO databank. **A** GSE160516 dataset displayed the expression levels of WSB1 in the heart of mice with IR treatment for different times or sham surgery. **B** The WSB1 levels in left ventricle tissues of rats with LAD ligation for 30 min followed with reperfusion for 2 days or 7 days, or sham treatment from GSE4105 dataset. (IR, ischemia–reperfusion; WSB1, WD repeat and SOCS box containing 1; LAD, left anterior descending; *p < 0.05, **p < 0.01, ***p < 0.001)

**Fig. 2:**
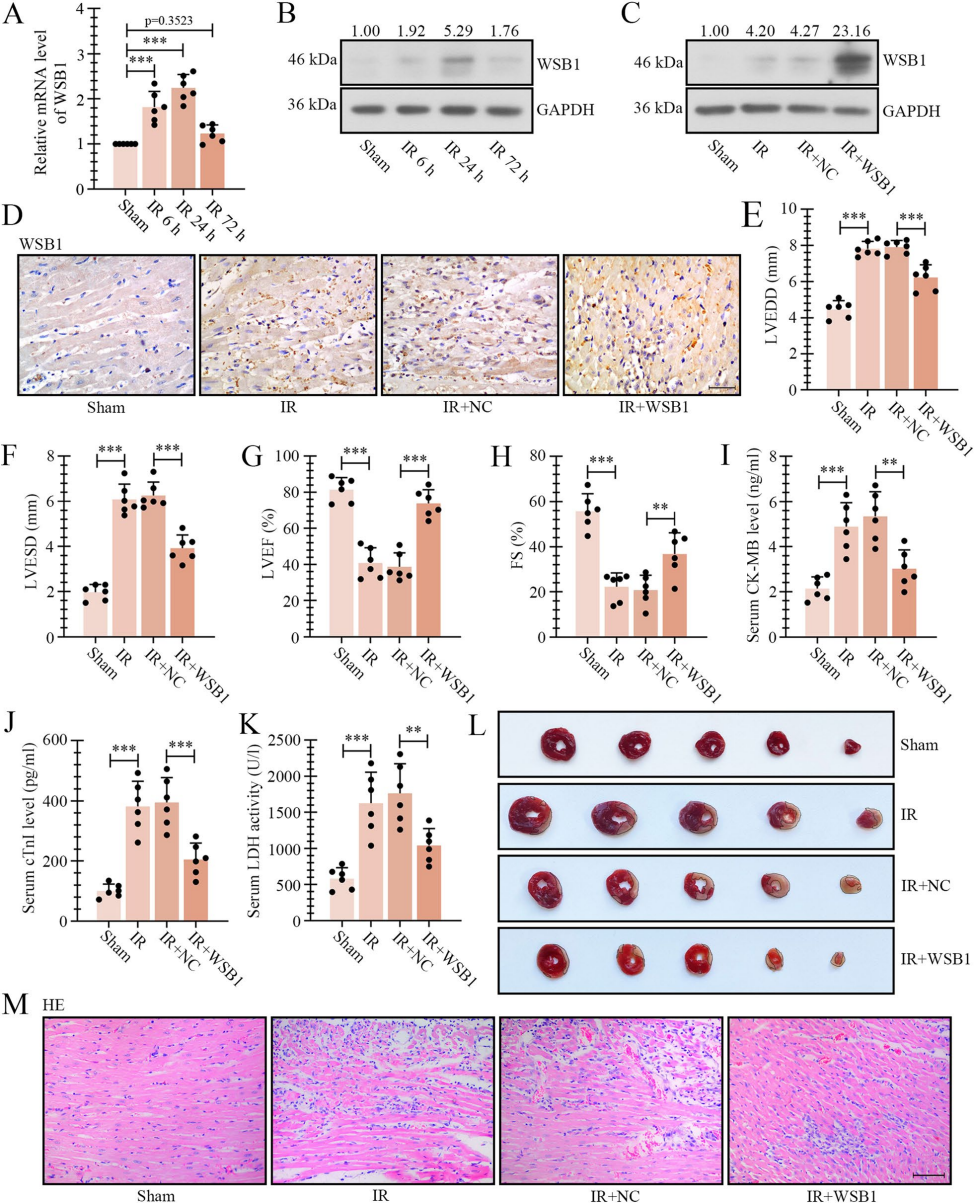
Overexpression of WSB1 alleviated myocardial IR-induced injury in rats. The rats were subjected to ligation of the LAD coronary artery for 45 min and reperfusion for 6 h, 24 h or 72 h to induce IR injury, and the mRNA (**A**) and protein levels of WSB1 (**B**) in myocardial infarct penumbra area were determined by real-time PCR and western blot. The AAV9 loaded with WSB1 coding sequence was delivered into rats via intravenous injection, and IR was performed two weeks later. The WSB1 expression was verified by western blot (**C**) and immunohistochemistry staining (**D**) in penumbra myocardial tissues (the scale bar represented 50 μm). The LVEDD (**E**), LVESD (**F**), LVEF (**G**) and FS (**H**) of rats after IR for 24 h were assessed with an animal ultrasonic instrument. The CK-MB (**I**) and cTnI levels in serum **J** were determined by ELISA. **K** The serum LDH activity. **L** The infarct size of rat hearts was displayed by TTC staining. **M** HE staining exhibited the pathological injury in ischemic penumbra area (the scale bar represented 100 μm). (IR, ischemia–reperfusion; WSB1, WD repeat and SOCS box containing 1; LAD, left anterior descending; LVEDD left ventricular end diastolic diameter; LVESD, left ventricular systolic diameter; LVEF, left ventricular fraction; FS, fractional shortening; LDH, Lactate dehydrogenase; TTC, Triphenyltetrazolium chloride; **p < 0.01, ***p < 0.001)

The analysis from GSE160516 dataset revealed that WSB1 was highly expressed in heart tissues of mice after myocardial IR for 6 h, 24 h and 72 h, compared with sham control (Fig. 1A). In GSE4105, the left ventricle tissues of rats displayed upregulation of WSB1 after myocardial IR for 2 or 7 days (Fig. 1B). In our study, the experimental myocardial IR was induced in rats by ligation of LAD coronary artery and blocking removing. Similar with the analyses from GEO datasets, real-time PCR and western blot revealed that the WSB1 mRNA and protein levels in penumbra area were upregulated at 6 h, 24 h and 72 h post IR surgery (Fig. 2A and B).

Our previous articles reported the beneficial effect of CG on cardiac function and cardiomyocyte survival, and the WSB1 expression was measured. As shown in Additional file 1: Fig. S1A, the increased WSB1 expression in IR-treated myocardial tissues was further elevated after CG application. These evidences suggested that WSB1 upregulation may be protective in myocardial IR.

### Overexpression of WSB1 alleviated IR-induced cardiac injury in rats.

In order to investigate the function of WSB1 in myocardial IR, the AAV9 loaded with WSB1 coding sequence was pre-delivered into IR rats via tail vein injection, and the overexpression of WSB1 was verified by western blot and immuohistochemistry staining (Fig. 2C and D). Echocardiograghy analysis revealed that IR led to cardiac damage, evidenced by increase of LVEDD and LVESD, and decrease of LVEF and FS, which were alleviated by WSB1 (Fig. 2E–H). The serum levels of myocardial injury markers, CK-MB and cTnI, were increased after IR treatment, while reduced in WSB1-loaded AAV9-infected rats (2I and 2 J). The LDH activity exhibited similar changes (Fig. 2K). The TTC staining of pale region notarized that IR-induced myocardial infarction was mitigated by WSB1 overexpression (Fig. 2L). HE staining revealed that the myocardial ischemic penumbra area from IR rats showed disorder of cell arrangement, myocardial borders breakage and inflammatory infiltration, compared with sham group, and these alterations were restrained after ectopic expression of WSB1 (Fig. 2M).

### WSB1 mitigated IR-induced cardiomyocyte apoptosis in rats

**Fig. 3:**
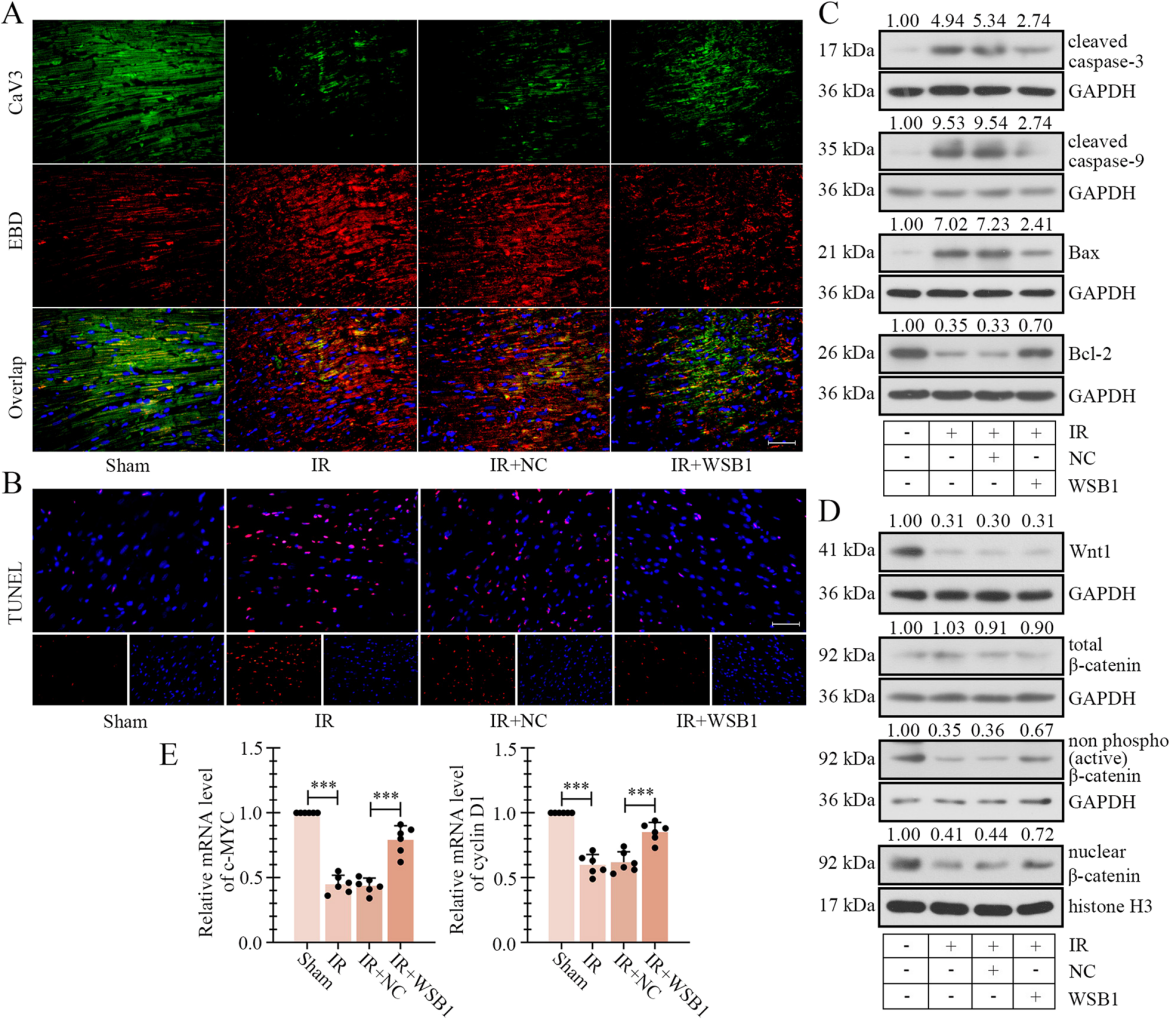
WSB1 inhibited IR-induced cell apoptosis in myocardial tissues. **A** CaV3 and EBD staining revealed the live and dead cells in ischemic penumbra area of rats with IR or/and WSB1 overexpression (CaV3 (green) indicated live cells and EBD (red) indicated dead cells, the scale bar represented as 50 μm). **B** TUNEL staining showed the apoptotic cells in penumbra area (the scale bar represented as 50 μm). **C** The levels of several apoptosis-related molecules, cleaved caspase-3, cleaved caspase-9, Bax and Bcl-2 were determined by western blot in penumbra area. **D** The levels of Wnt1, total β-catenin, non phosphor (active) β-catenin and nuclear β-catenin in penumbra area. **E** The mRNA levels of β-catenin targets, c-MYC and cyclin D1 in penumbra area. (IR, ischemia–reperfusion; WSB1, WD repeat and SOCS box containing 1; CaV3, Caveolin-3; EBD, Evans blue dye; ***p < 0.001)

Cardiomyocyte necrosis and apoptosis are vital damage of myocardial IR. The CaV3/EBD staining showed that in infarct penumbra area, the numbers of living cells (CaV3-positive) was drastically reduced and dead cell numbers (EBD-positive) rapidly increased after IR treatment, while these alterations were partially reversed by overexpression of WSB1 (Fig. 3A). TUNEL assay demonstrated that IR-induced cell apoptosis was alleviated by WSB1 in penumbra area (Fig. 3B). The levels of pro-apoptotic molecules, cleaved caspase-3, cleaved caspase-9 and Bax, were increased, and the level of anti-apoptotic molecule Bcl-2 was decreased in ischemic penumbra area of IR rats. These alterations were ameliorated by WSB1 (Fig. 3C), which were consistent with the TUNEL results. Additionally, the levels of Wnt1, active β-catenin and nuclear β-catenin were decreased in penumbra area of IR, indicating the deactivation of Wnt/β-catenin signaling. After WSB1 overexpression, the activation and nuclear translocation of β-catenin were enhanced, but the Wnt1 expression was not influenced (Fig. 3D). Similarly, the mRNA levels of β-catenin downstream targets, c-MYC and cyclin D1 was also promoted by WSB1 (Fig. 3E). The above results demonstrated that WSB1 restrained IR-induced cell apoptosis in penumbra area, and β-catenin signaling may be involved.

### WSB1 restrained OGD/R-induced apoptosis of myocardial cells in vitro

**Fig. 4:**
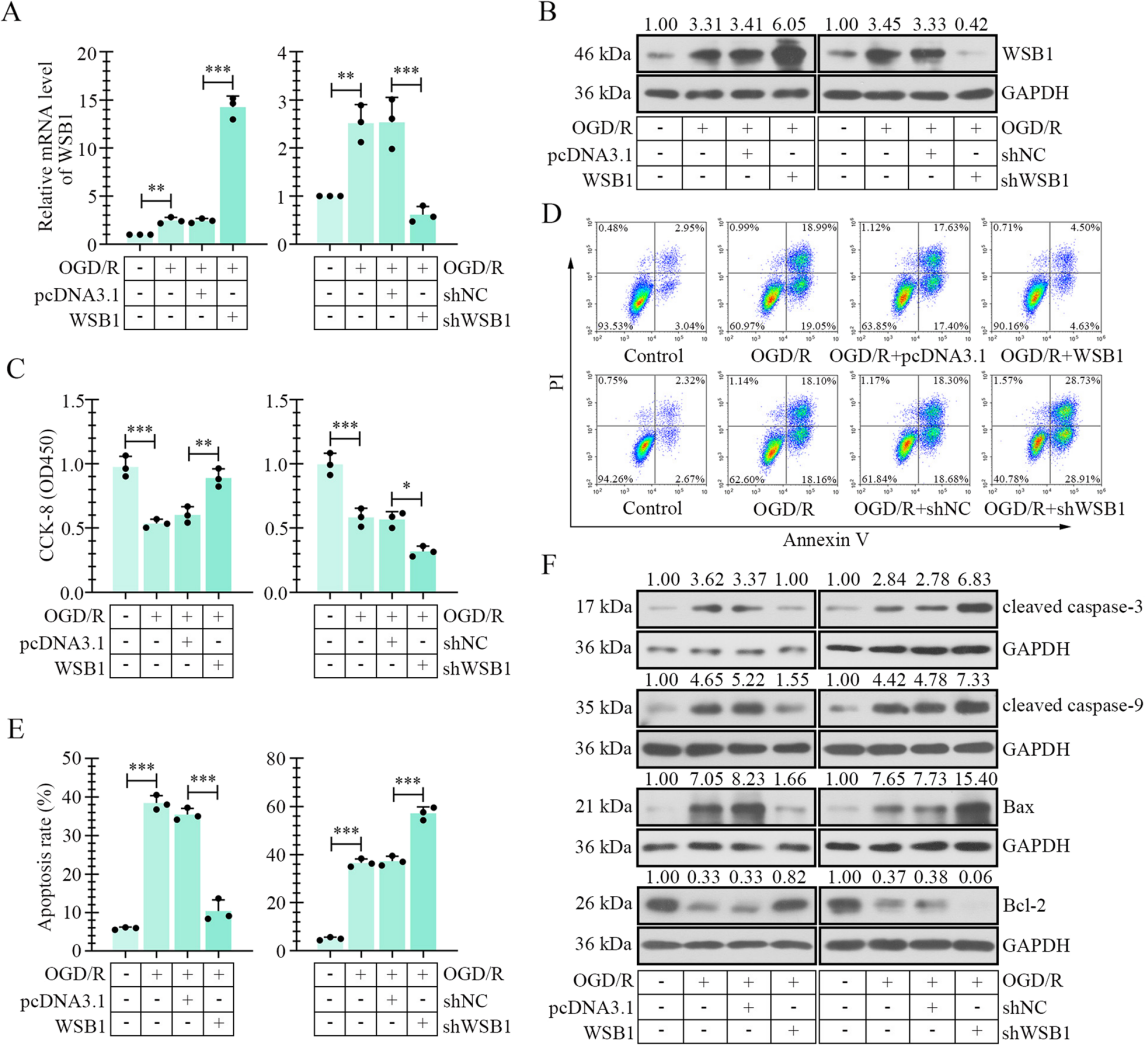
WSB1 ameliorated OGD/R-caused apoptosis in myocardial cells. The mRNA **A** and protein levels of WSB1 **B** in myocardial cells with OGD/R treatment and WSB1 overexpression/knockdown were assessed with real-time PCR and western blot. **C** CCK-8 assay was used to measure the viability of myocardial cells. **D** and **E** Flow cytometry was performed to evaluate the apoptosis of myocardial cells. **F** The levels of apoptosis-related molecules, cleaved caspase-3, cleaved caspase-9, Bax and Bcl-2 were detected by western blot in myocardial cells with OGD/R treatment and ectopic expression or silencing of WSB1. (OGD, oxygen-sugar deprivation; WSB1, WD repeat and SOCS box containing 1; PI, propidium iodide; *p < 0.05, **p < 0.01, ***p < 0.001)

To further investigate the function details of WSB1 in myocardial tissues, the immortalized rat myocardial cell line, H9c2, was cultured and subjected to an OGD/R treatment to mimic heart IR. The results from real-time PCR and western blot revealed that OGD/R administration led to upregulated expression of WSB1 at transcriptional and translational levels (Fig. 4A, B, Additional file 1: Fig. S1C and S1D). The CG application induced a further upregulation of WSB1 (Additional file 1: Fig. S1C and S1D), which was consistent with the results in vivo. The overexpression and silencing of WSB1 were realized by transfection of overexpression or knockdown plasmid in H9c2 cells (Fig. 4A, B). CCK-8 assay exhibited that WSB1 promoted the viability of H9c2 cells, and WSB1 knockdown suppressed its viability under OGD/R condition (Fig. 4C). Flow cytometry detection suggested that OGD/R-induced apoptosis of H9c2 cells was attenuated by WSB1 overexpression and aggravated by WSB1 silencing (Fig. 4D, E). The OGD/R-induced elevation of levels of cleaved caspase-3/9 and Bax, and decline of Bcl-2 level, were abolished by enhanced expression of WSB1 and exacerbated by its knockdown (Fig. 4F), which supported the results of flow cytometry.

### WSB1 activated β-catenin signaling by mediating the ubiquitination of GSK3β

**Fig. 5:**
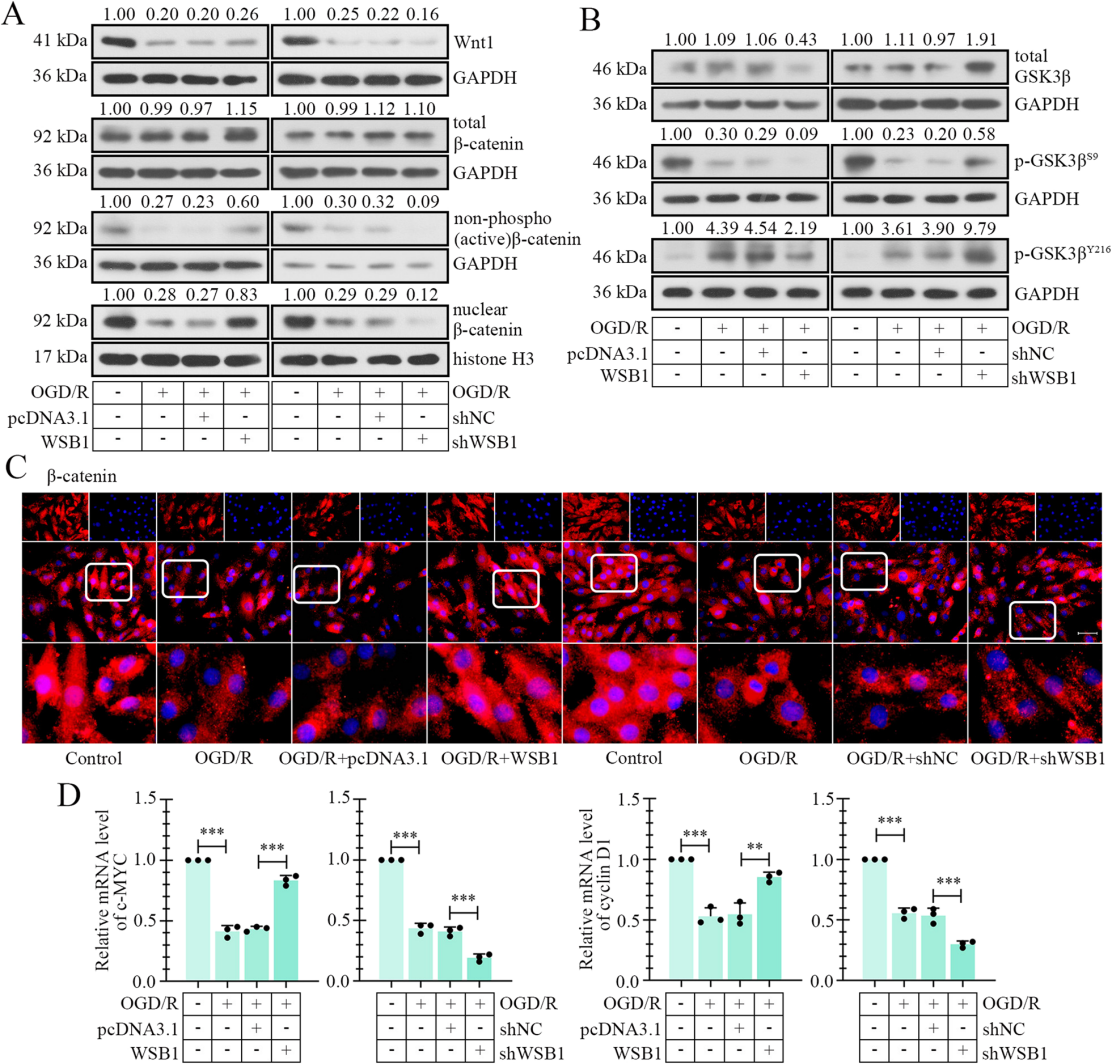
WSB1 resulted in activation of β-catenin signaling in myocardial cells. **A** The levels of Wnt1, total β-catenin, non phosphor (active) β-catenin and nuclear β-catenin in myocardial cells. **B** The total GSK3β, p-GSK3β^S9^ and p-GSK3β.^Y216^ levels in myocardial cells after OGD/R administration and WSB1 overexpression/silencing. **C** The distribution of β-catenin in myocardial cells was indicated with immunofluorescence staining (the scale bar represented as 50 μm). **D** The mRNA levels of c-MYC and cyclin D1. (OGD, oxygen-sugar deprivation; WSB1, WD repeat and SOCS box containing 1; GSK3β, glycogen synthase kinase 3 beta; **p < 0.01, ***p < 0.001)

**Fig. 6:**
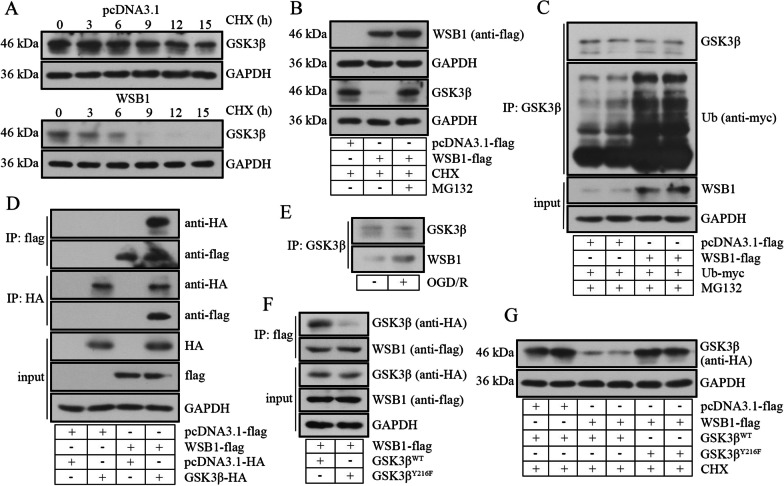
WSB1 suppressed GSK3β via ubiquitination-mediated degradation of in myocardial cells. **A** The level of WSB1 in myocardial cells with CHX administration for 0 h, 3 h, 6 h, 9 h, 12 h or15 h and WSB1 overexpression. **B** The exogenous WSB1 and cellular GSK3β levels in myocardial cells with WSB1 overexpression and application of CHX or/and MG132. **C** The ubiquitin content bound by GSK3β antibody was determined by co-IP in myocardial cells with/without WSB1 overexpression. **D** Co-IP was carried out to confirm the combination of exogenous WSB1 and GSK3β in myocardial cells. **E** The binding between endogenous WSB1 and GSK3β was verified by co-IP in myocardial cells with OGD/R treatment. **F** The binding of WSB1 to wild type or mutant GSK3β was detected. (**G**) The wild type or mutant GSK3β level was examined after WSB1 overexpression and CHX administration. (OGD, oxygen-sugar deprivation; WSB1, WD repeat and SOCS box containing 1; GSK3β, glycogen synthase kinase 3 beta; WT, wild type; CHX, cycloheximide)

The activity of β-catenin signaling was assessed in H9c2 cells. Western blot and immunofluorescence staining results displayed the reduction of active β-catenin, and the nuclear translocation of β-catenin, as well as the Wnt1 expression and transcription of c-MYC and cyclin D1 in hypoxic cells, indicating the deactivation of Wnt/β-catenin signaling in H9c2 cells (Fig. 5A–E). WSB1 upregulation caused the activation and nuclear distribution of β-catenin, as wells as the transcription of downstream target genes, but not Wnt1 expression, indicating that WSB1 regulating the β-catenin signaling was not mediated by Wnt1. Additionally, we focused on another key mediator GSK3β. It was reported that the phosphokinase activity of GSK3β was activated by phosphorylation at Y216, and inhibited by phosphorylation at S9 (Varisli et al. 2015), so the levels of p-GSK3β^Y216^ and p-GSK3β^S9^ in H9c2 was determined. As shown in Fig. 5B, with the activation of β-catenin signaling, the level of p-GSK3β^Y216^ was reduced, consistent with total GSK3β. While, the level of p-GSK3β^S9^ decreased after OGD/R treatment, but not increased along with the WSB1 overexpression and activation of β-catenin signaling. Thereafter, the effect of WSB1 on GSK3β level was examined. As exhibited in Fig. 6A, the degradation rate of GSK3β was accelerated by WSB1 with CHX administration. However, the effect of WSB1 was abolished when a proteasome inhibitor MG132 was applied (Fig. 6B), suggesting that GSK3β was degraded by ubiquitin–proteasome pathway. Next, co-IP results revealed that WSB1 promoted the ubiquitination of GSK3β (Fig. 6C). The binding between WSB1 and GSK3β was confirmed in H9c2 cells, and this binding was enhanced under OGD/R condition (Fig. 6D, E). Considering that the phosphorylation of Y216 was crucial for phosphokinase activity of GSK3β, an Y216F mutant with phosphorylation defectiveness was used. The results revealed that WSB1 bound to GSK3β^WT^, but not Y216F mutant (Fig. 6F), and WSB1 almost did not affect GSK3β^Y216F^ degradation (Fig. 6G). The results in this section suggested that WSB1 may activate β-catenin signaling by binding and degrading GSK3β in myocardial cells, and this binding depended on the phosphorylation of GSK3β at Y216.

### The effects of WSB1 on myocardial cells were abrogated by inhibition of β-catenin signaling

**Fig. 7:**
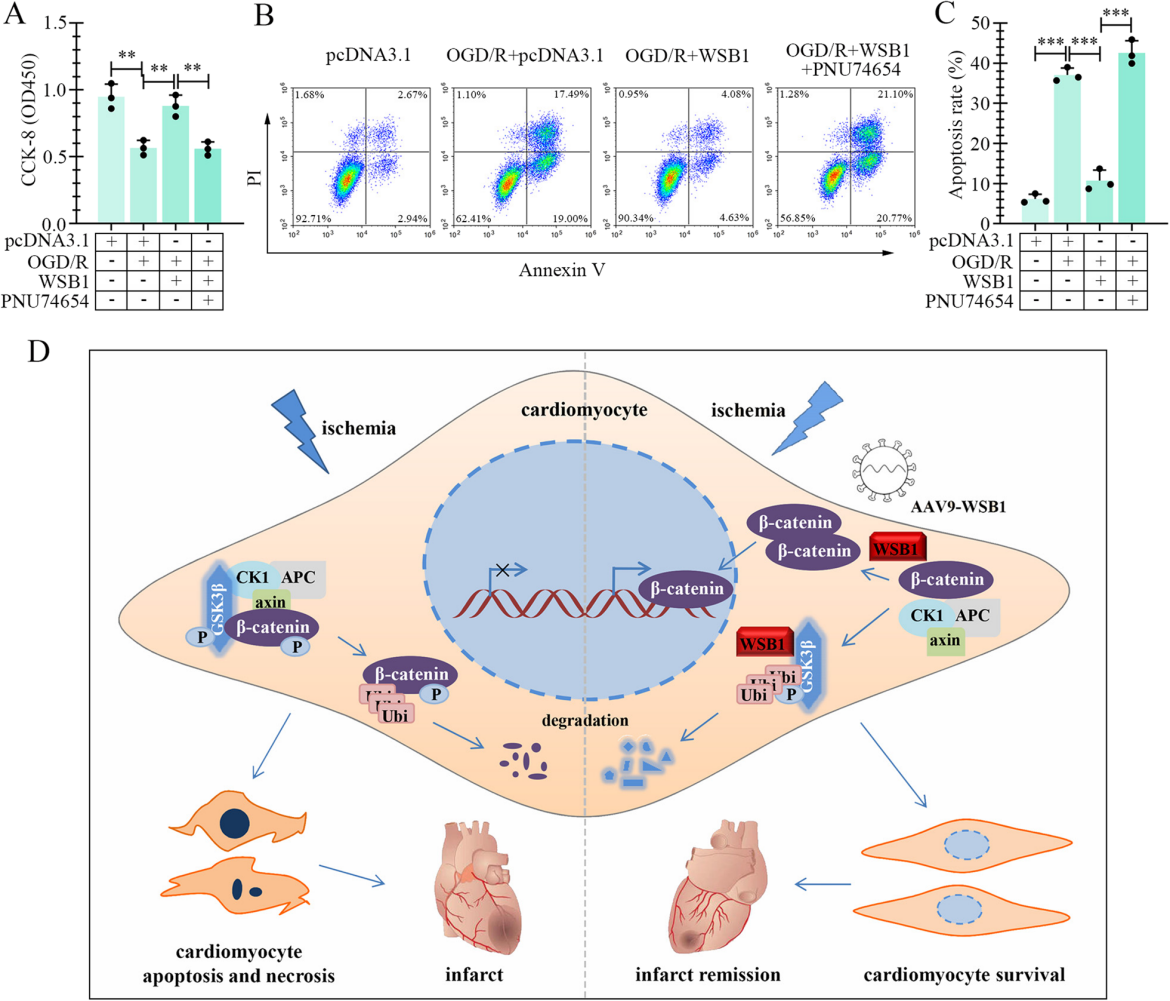
The effect of WSB1 on apoptosis was reversed by an inhibitor of β-catenin signaling. **A** The viability of myocardial cells with WSB1 overexpression and treatment of OGD/R and an inhibitor of β-catenin, PNU74654 was measured by CCK-8 assay. **B** and **C** The apoptosis of myocardial cells. **D** WSB1 played a protective role in cardiomyocytes by mediating the ubiquitination and degradation of GSK3β and inactivating β-catenin signaling under an ischemic condition. (OGD, oxygen-sugar deprivation; WSB1, WD repeat and SOCS box containing 1; PI, propidium iodide; GSK3β, glycogen synthase kinase 3 beta; **p < 0.01, ***p < 0.001)

The above results demonstrated that WSB1 mitigated OGD/R-induced apoptosis of myocardial cells, and activated β-catenin signaling. Here, an inhibitor of β-catenin signaling, PNU74654, was used. As displayed in Fig. 7A, the viability of H9c2 cells was inhibited by OGD/R, promoted by WSB1 overexpression, and suppressed by PNU74654. Similarly, OGD/R-induced apoptosis of H9c2 cells was reduced by WSB1, and increased by PUN74654 (Fig. 7B and C).

## Discussion

In this study, we demonstrated that WSB1, which was increased in myocardial tissues and cardiomyocytes with ischemic stimulation, alleviated IR or OGD/R-induced cell death and cardiac function injury, and activated β-catenin signaling via mediating ubiquitination and proteasomal degradation of GSK3β (Fig. 7D).

WSB1 is a deeply conserved protein in multiple species. It contains several WD repeats in N-terminal and a suppressor of cytokine signaling (SOCS) box in C-terminal (Vasiliauskas et al. 1999). The WD domain defines a common structure in eukaryote proteins, and the SOCS box interacts with the components of E3 ubiquitin ligase complex, such as VHL, Elongin B and C (Kibel et al. 1995; Zhang et al. 1999). WSB1 acts as an E3 ligase via the SOCS box, which determines the substrate specificity (Linossi and Nicholson 2012). A congener WSB2 contains similar WD repeats and SOCS box. WSB1 and WSB2 have same cancer-promoting roles (Zhang et al. 2019; Archange et al. 2008). Our data demonstrated that WSB1 activated β-catenin signaling, but this regulation was mediated by GSK3β, but not wnt. A previous article reported that WSB2 activated Wnt/β-catenin signaling (Wei et al. 2020). However, we found that they just demonstrated the promoting effect of WSB2 on levels of c-MYC and β-catenin, but not that of wnt, which was similar with our data. Additionally, this regulation was also reported by Gao et al*.* in hepatocellular carcinoma cells (Gao et al. 2022). However, WSB2 has not been reported to act as an E3 ligase. It is currently unclear how WSB2 regulates β-catenin signaling, and whether it regulates GSK3β like WSB1 does.

GSK3β is a serine-threonine kinase catalyzing phosphorylation of lots of substrates, such as β-catenin, c-Jun, tau and NF-ATc1 (Dajani et al. 2003; Boyle et al. 1991; Cho and Johnson 2004; Beals et al. 1997). GSK3β-mediated ubiquitination of β-catenin is achieved by assembling the destruction complex with axin, APC and CK1. CK1 mediates the phosphorylation of β-catenin at S45, which creates a priming site for GSK3β. Next, GSK3β catalyzes the phosphorylation at T41, S37 and S33, among which S33 and S37 phosphorylation provides docking sites for the E3 ubiquitin ligase, β-transducing repeat-containing protein (β-TrCP), followed with proteasomal degradation of β-catenin (Shah and Kazi 2022; Liu et al. 2002). As the core protein, axin mediates the whole assembly of the destruction complex. GSK3β phosphorylates axin and APC, to further enhance their affinity to β-catenin (Stamos and Weis 2013). In the presence of canonical wnt ligand, the axin is recruited to membrane receptor, and the destruction complex dissolves. The non-phosphorylated β-catenin is released, accumulates and translocates into nucleus to activate the transcription of target genes (Chiurillo 2015).

Although all components of the destruction complex are responsible for degradation of β-catenin, the GSK3β-mediated phosphorylation is most crucial. The phosphokinase activity of GSK3β is activated by phosphorylation at Y216, and inhibited by phosphorylation at S9 (Varisli et al. 2015). In our study, ischemia induced reduction of non-phosphorylated β-catenin (no phosphorylation at T41, S33 or S37) level and the elevation of Y216 phosphorylated GSK3β level, suggesting the inactivation of β-catenin in myocardial cells. However, the increase of total GSK3β after ischemia stimulation was mild, while the p-GSK3β^S9^ and p-GSK3β^Y216^ levels changed dramatically, suggesting that ischemia treatment may primarily regulated the kinase activity of GSK3β, not its expression. However, we demonstrated that WSB1 bound to GSK3β to mediate its ubiquitination and degradation, and this binding depended on phosphorylation at Y216. Similar results were observed in another paper (Gao et al. 2015), which reported that phosphorylation at Y216 was essential for β-TrCP-mediated ubiquitination of GSK3β in cancer cells. These evidences demonstrated that phosphorylation at Y216 was necessary for both kinase activity and ubiquitination, which interestingly suggested that the active form of GSK3β was unstable. Moreover, the phosphorylation of GSK3β at Y216 is catalyzed by itself (Cole et al. 2004). We hypothesized that GSK3β precisely regulated β-catenin by controlling its own kinase activity and stability. Additionally, the binding between WSB1 and GSK3β was augmented under a hypoxic condition, which may be due to the enhanced Y216 phosphorylation level of GSK3β induced by ischemia.

WSB1 was increased in heart penumbra area of IR rats, and AAV9-mediated WSB1 upregulation in advance prevented IR-induced myocardial injury, suggesting that the increase of WSB1 in ischemic heart was protective. Our results firstly revealed that the enhanced expression of WSB1 was beneficial for maintaining cardiac function and sustaining cardiomyocyte survival in rats after IR stimulation. In addition, oxidative stress reaction after IR caused severe injury in heart. Oxidative stress is generally caused by imbalance between toxic reactive oxygen species (ROS) production and scavenging, leading to the accumulation of peroxides, superoxides and hyperoxide (Curtsinger et al. 1983). ROS is primarily produced during aerobic metabolism in mitochondria. Maintaining cardiac function requires significant amounts of ATP, and 30–40% of cardiomyocyte volume is comprised of mitochondrial (Bugger and Pfeil 2020; Barth et al. 1992). The ischemia and reperfusion cause a burst of ROS, which results in damage of organelle and macromolecules via many pathways due to the exhaustion of reductases (Xiang et al. 2021). Therefore, inhibiting the generation ROS or accelerating its disintegration could alleviate IR damage in myocardial cells. For instance, allopurinol, a xanthine oxidase inhibitor that could suppress purine-derived ROS production, was reported to reduce infarct size, improve ventricular function and prevent arrhythmia in dogs (Stewart et al. 1985). Nuclear factor erythroid-related factor 2 (Nrf2) promotes the expression of lots of antioxidases, such as heme oxygenase 1 (HO-1) and superoxide dismutase 1 (SOD), and activated Nrf2 was beneficial for limiting infarct size and maintaining cardiac function in rats (Kaspar et al. 2009; Yu et al. 2016). Wnt/β-catenin signaling also plays a protective role in myocardial IR by reducing the ROS level in mice (Shen et al. 2022). WSB1 was reported to suppress hypoxia-induced cell apoptosis in tumor cells (Kim et al. 2015; Tong et al. 2013), and our study demonstrated that WSB1 restrained IR-caused cardiac dysfunction and cardiomyocyte death, but its effects on ROS or oxidative stress reaction remained unclear.

It is well known that IR may induce many types of damage, including cardiac dysfunction, inflammation, oxidative stress reaction, autophagy, apoptosis, ferroptosis and pyrotosis, and large infarct and a large number of myocardial cell death are the deadliest. All treatments for myocardial IR aim to maintain the normal function of heart and the survival of myocardial cells as much as possible. Our study demonstrated that the overexpression of WSB1 mitigated IR-induced cardiac dysfunction, myocardial injury and cardiomyocyte death. In this situation, the effect of WSB1 on oxidative stress reaction does not seem to enhance its cardiac protective function. However, it is also meaningful for investigating the function mechanism of WSB1. In our previous reports, CG administration ameliorated myocardial IR-induced malondialdehyde elevation and promoted the SOD content in rats (Ren et al. 2016), and WSB1 was increased after CG treatment and play a cardioprotective role. Combined with the protective effects of WSB1 on hypoxic tumor cells, we hypothesized that WSB1 may inhibit IR-induced ROS burst and oxidative stress reaction, which needed to be confirmed by further investigation.

Additionally, WSB1 was upregulated after IR-treated rats and myocardial cells, and the overexpression of WSB1 played a protective role in hypoxic condition, suggesting that the upregulation in models may be reparative, and the organisms exerted self-protection mechanisms when harmed. Maintaining and even exacerbating the high expression of WSB1 may be beneficial for myocardial ischemic patients. So their agonists may be promising. On the other hand, as an E3 ligase, WSB1 acts by mediating the ubiquitination and degradation of substrates, such as GSK3β. Therefore, developing the inhibitors of WSB1 substrates is also worth looking forward to.

The roles of GSK3β in myocardial ischemia have been reported in previous papers. Several articles revealed the protective effects of GSK3β inhibitors in myocardial IR (Kim et al. 2022; Zhai et al. 2011). After reperfusion, the excessive ROS leads to phosphorylation of GSK3β at Y216, and phosphorylated GSK3β inhibits mitochondrial permeability transition pores (MPTP) opening (Ghaderi et al. 2017). MPTP is a nonspecific channel in the mitochondrial inner membrane which allows for the passing of molecules less than 1.5 kDa in size. Once MPTP opens, the mitochondrial membrane potential is lost after 10 min (Kinnally et al. 2011). GSK3β inactivation significantly inhibits MPTP opening, as well as ROS increase and calcium in reperfusion state (Linseman et al. 2004). These evidences demonstrate that activated GSK3β is harmful during myocardial IR. Our results revealed that WSB1 played a protective role in myocardial IR by mediating ubiquitination and degradation of GSK3β with Y216 phosphorylation, and we supposed that WSB1 might suppress the ROS production and mitochondrial damage.

In conclusions, we demonstrated that WSB1 was highly expressed in myocardium of rats after IR stimulation. The overexpression of WSB1 alleviated IR or OGD/R-induced cardio dysfunction and myocardial cell death by mediating ubiquitination of GSK3β activating β-catenin signaling. These findings may provide novel insights for preventive or protective strategies in clinical myocardial ischemia treatment.

### Supplementary Information

**Additional file 1: Figure S1. **WSB1 was increased after CG treatment in myocardial tissues and myocardial cells with IR. The mRNA (**A**) and protein levels (**B**) in myocardial tissues of rats with IR and CG treatment. **C** and **D** The WSB1 levels in myocardial cells with application of OGD/R and CG. (OGD, oxygen-sugar deprivation; IR, ischemia-reperfusion; WSB1, WD repeat and SOCS box containing 1; CG, calycosin-7-O-β-D-glucoside; *p<0.05, ***p<0.001).

## Data Availability

Data will be made available on request.
