# Supplementary figures and images for: WSB1, as an E3 ligase, restrains myocardial ischemia–reperfusion injury by activating β-catenin signaling via promoting GSK3β ubiquitination
Source: Mol Med. 2024 Feb 23;30:31. doi: 10.1186/s10020-024-00800-3 (PMC10893653; doi:10.1186/s10020-024-00800-3)

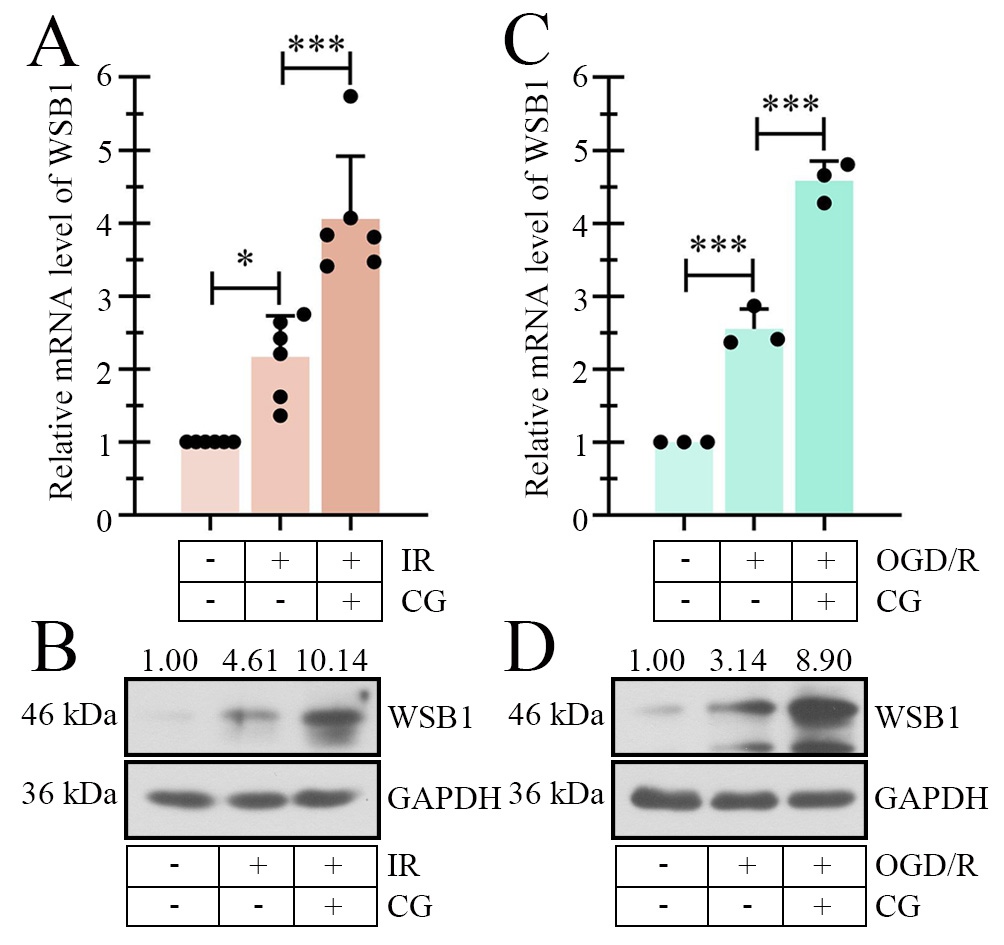

Supplement: Supplementary file 1 — Additional file 1: Figure S1. WSB1 was increased after CG treatment in myocardial tissues and myocardial cells with IR. The mRNA (A) and protein levels (B) in myocardial tissues of rats with IR and CG treatment. C and D The WSB1 levels in myocardial cells with application of OGD/R and CG. (OGD, oxygen-sugar deprivation; IR, ischemia-reperfusion; WSB1, WD repeat and SOCS box containing 1; CG, calycosin-7-O-β-D-glucoside; *p<0.05, ***p<0.001). [file 10020_2024_800_MOESM1_ESM.jpg]
